# Supplementary material for: Phylogeography of the widespread Caribbean spiny orb weaver Gasteracantha cancriformis
Source: PeerJ. 2020 Apr 30;8:e8976. doi: 10.7717/peerj.8976 (PMC7196328; doi:10.7717/peerj.8976)
Supplement: Supplemental Information 5 — All samples from Colombia and Brazil were acquired from GenBank and originally made accessible by Saldago-Roa et al. (2018). [file peerj-08-8976-s005.docx]

| Locality | Barcode ID | COI | 16S | ITS2 | Region | Latitude | Longitude | Color | # of spines | Genetic cluster | COI haplotype |
| --- | --- | --- | --- | --- | --- | --- | --- | --- | --- | --- | --- |
| Cuba | CU00784364 | X | X | X | ViÐales | 22.62109 | -83.73834 | yellow | 4 | 1 | hap1 |
|  | CU00787752 | X |  |  | El Yunque | 20.33178 | -74.56919 | yellow | 4 | 2 | hap2 |
|  | CU00787789 | X |  |  | El Yunque | 20.33178 | -74.56919 | yellow | 4 | 1 | hap3 |
|  | CU00787790 | X |  |  | El Yunque (Baraca) | 20.35756 | -74.50514 | yellow | 4 | 1 | hap4 |
| Hispaniola | HI00782393 | X |  |  | Parque Nacional del Este | 18.355536 | -68.61825 |  |  |  |  |
|  | HI00782411 | X |  |  | Parque Nacional del Este | 18.355536 | -68.61825 |  |  |  |  |
|  | HI00782516 | X |  | X | Parque Nacional Cotubanama | 18.373354 | -68.819834 | black/red | 4 | 1 | hap7 |
|  | HI00784368 | X |  |  | Parque Nacional Cotubanama | 17.68333 | -71.26667 |  |  |  |  |
|  | HI00784515 | X |  |  | Parque Nacional Cotubanama | 18.3816 | -68.8017 | black/red | 4 | 2 | hap5 |
|  | HI00784655 | X |  |  | Puerto Plata | 19.789 | -70.717 | black/red | 4 | 2 | hap8 |
|  | HI00784859 | X |  |  | Puerto Plata | 19.789 | -70.717 | white | 4 | 2 | hap9 |
|  | HI00784862 | X |  |  | Villa Elisa | 19.08013 | -69.4649 |  |  |  |  |
|  | HI00784870 | X | X | X | Villa Elisa | 19.08013 | -69.4649 |  |  |  |  |
|  | HI00784960 | X |  | X | Parque Nacional Cotubanama | 18.32902 | -68.80995 |  |  |  |  |
|  | HI00784987 | X |  |  | Villa Elisa | 17.68333 | -71.26667 |  |  |  |  |
|  | HI00784988 | X |  |  | Puerto Plata | 19.789 | -70.717 |  |  |  |  |
|  | HI00785091 | X |  |  | Parque Nacional Cotubanama | 18.35554 | -68.61825 | black/red | 4 |  | hap6 |
|  | HI00785150 | X |  |  | Villa Elisa | 17.68333 | -71.26667 |  |  |  |  |
|  | HI00785264 | X | X |  | Parque Nacional del Este | 18.355536 | -68.61825 |  |  |  |  |
|  | HI00785327 | X | X | X | Willy | 19.07796 | -69.46635 | black/red | 4 | 3 | hap13 |
|  | HI00785333 | X | X | X | Parque Nacional del Este | 18.35554 | -68.61825 |  |  |  |  |
|  | HI00785345 | X |  |  | Willy | 19.07796 | -69.46635 | black/red | 4 | 3 | hap13 |
|  | HI00785382 | X |  |  | Puerto Plata | 19.789 | -70.717 | yellow | 4 | 2 | hap9 |
|  | HI00785414 | X |  |  | Puerto Plata | 19.789 | -70.717 | white | 4 | 2 | hap8 |
|  | HI00785442 | X |  |  | Puerto Plata | 19.789 | -70.717 | white | 4 |  |  |
|  | HI00785458 | X |  |  | Puerto Plata | 19.789 | -70.717 | yellow | 4 | 2 | hap8 |
|  | HI00785460 | X |  |  | Puerto Plata | 19.789 | -70.717 | black/red | 4 | 2 | hap9 |
|  | HI00785570 | X | X | X | Willy | 19.08013 | -69.4649 | black/red | 4 | 2 | hap14 |
|  | HI00785581 | X | X | X | Puerto Plata | 19.789 | -70.717 | white | 4 | 2 | hap8 |
|  | HI00785620 | X |  |  | Puerto Plata | 19.789 | -70.717 | white | 4 | 2 | hap9 |
|  | HI00785629 | X |  |  | Parque Nacional Cotubanama | 19.07796 | -69.46635 | black/red | 4 | 3 | hap13 |
|  | HI00785630 | X |  |  | Parque Nacional del Este | 18.35554 | -68.61825 | black/red | 4 | 2 | hap5 |
|  | HI00785637 | X |  |  | Puerto Plata | 19.789 | -70.717 | black/red | 4 |  |  |
|  | HI00785653 | X | X |  | Willy | 19.07796 | -69.46635 | yellow | 4 | 3 | hap13 |
|  | HI00785662 | X |  |  | Puerto Plata | 19.789 | -70.717 | white | 4 |  |  |
|  | HI00785720 | X |  |  | Puerto Plata | 19.789 | -70.717 | black/red | 4 |  |  |
|  | HI00785777 | X |  |  | Puerto Plata | 19.789 | -70.717 | white | 4 |  |  |
|  | HI00787054 | X | X | X | Lago Enriquillo (Rabo de Gato) | 18.4788 | -71.59613 | yellow | 4 | 2 | hap15 |
|  | HI00787065 | X |  |  | Lago Enriquillo (Rabo de Gato) | 18.4788 | -71.59613 |  |  |  |  |
|  | HI00787198 | X |  |  | Rabo de Gato | 18.3159 | -71.5792 |  |  |  |  |
|  | HI00787228 | X |  |  | Rabo de Gato | 18.3159 | -71.5792 | black | 4 |  |  |
| Jamaica | JA00003378A | X |  |  | Coopers Pen (Cockpit) | 18.45892 | -77.6058611 | white | 4 |  |  |
|  | JA00003414A | X | X | X | Coopers Pen (Cockpit) | 18.45892 | -77.6058611 | black | 4 | 1 | hap17 |
|  | JA00003433A | X | X | X | Port Royal | 17.85133 | -76.9693889 | white | 4 | 1 | hap18 |
|  | JA00003455A | X | X |  | Drapers | 18.17111 | -76.4217778 |  |  |  |  |
|  | JA00003523A | X |  |  | Coopers Pen (Cockpit) | 18.45892 | -77.6058611 | white | 4 |  |  |
|  | JA00003528A | X | X | X | Coopers Pen (Cockpit) | 18.45892 | -77.6058611 |  |  |  |  |
|  | JA00003589A | X |  |  | Port Royal | 17.85133 | -76.9693889 | Black/yel | 4 | 1 | hap21 |
|  | JA00003644A | X | X |  | Port Royal | 17.85133 | -76.9693889 | black | 4 |  |  |
|  | JA00003657A | X |  |  | Port Royal | 17.85133 | -76.9693889 | white | 4 | 1 | hap18 |
|  | JA00004115A | X |  |  | Holywell Park (BlueMountains) | 18.08903 | -76.72765 | black | 4 | 1 | hap22 |
|  | JA00004145A | X | X | X | Millbank | 18.01336 | -76.37975 | black | 4 | 1 | hap23 |
|  | JA00004146A | X |  |  | Hagley Gap | 18.02389 | -76.6392556 | black | 4 | 1 | hap19 |
|  | JA00004168A | X |  |  | Millbank | 18.01336 | -76.37975 | black | 4 | 1 | hap24 |
|  | JA00004176A | X |  |  | Hagley Gap | 18.02389 | -76.6392556 |  |  |  |  |
|  | JA00004204A | X |  |  | Millbank | 18.01336 | -76.37975 | black | 4 | 1 | hap23 |
|  | JA00004211A | X |  |  | Drapers | 18.17111 | -76.4217778 | white | 4 | 1 | hap19 |
|  | JA00004225A | X |  |  | Hagley Gap | 18.02389 | -76.6392556 | black | 4 | 1 | hap19 |
|  | JA00004290A | X |  |  | Hagley Gap | 18.02389 | -76.6392556 |  |  |  |  |
|  | JA00004309A | X |  |  | Drapers | 18.17111 | -76.4217778 | white | 4 | 1 | hap19 |
|  | JA00004453A | X |  |  | Drapers | 18.17111 | -76.4217778 |  |  |  |  |
| Lesser Antilles | LA00000127A | X |  |  | Nevis | 17.14145 | -62.57784 |  |  |  |  |
|  | LA00000131A | X | X | X | Nevis | 17.14162 | -62.59573 | white | 6 |  |  |
|  | LA00000326A | X |  |  | Nevis | 17.14162 | -62.59573 | white | 6 |  |  |
|  | LA00000408A | X |  |  | Nevis | 17.11804 | -62.58705 | white | 6 | 3 | hap28 |
|  | LA00000484A | X | X | X | Martinique | 14.739 | -61.10097 |  |  |  |  |
|  | LA00000498A | X | X |  | Nevis | 17.11804 | -62.58705 | white | 6 | 3 | hap27 |
|  | LA00001080A | X |  |  | Gustavia (Anguilla) | 17.91924 | -62.86366 | white | 6 | 3 | hap29 |
|  | LA00001163A | X | X | X | Oranjestad (Nevis) | 17.47897 | -62.97165 | white | 6 | 3 | hap29 |
|  | LA00001232A | X |  |  | Guadeloupe | 16.43977 | -61.42054 | white | 6 | 3 | hap30 |
|  | LA00001297A | X |  |  | Saint-Martine (Anguilla) | 18.07638 | -63.04991 | white | 6 | 3 | hap29 |
|  | LA00001301A | X |  |  | Gustavia (Anguilla) | 17.91924 | -62.86366 | white | 6 | 3 | hap29 |
|  | LA00001304A | X |  |  | Guadeloupe | 16.48456 | -61.43409 | white | 6 | 3 | hap30 |
|  | LA00001496A | X |  |  | Grand-Bourg (Guadeloup) | 15.91641 | -61.24602 |  |  |  |  |
|  | LA00001506A | X | X | X | Grand-Bourg (Guadeloup) | 15.91641 | -61.24602 | black | 6 | 3 | hap30 |
|  | LA00001654A | X |  |  | St Lucia | 13.86685 | -60.91768 | black | 6 | 3 | hap31 |
|  | LA00001791A | X |  |  | St Lucia | 13.86685 | -60.91768 | black | 6 | 3 | hap31 |
|  | LA00787620 | X |  |  | Gustavia (Anguilla) | 17.91924 | -62.86366 |  |  |  |  |
|  | LA00787984 | X |  |  | Gustavia (Anguilla) | 17.88905 | -62.82259 |  |  |  |  |
|  | LA00787994 | X |  |  | Guadeloupe | 16.43977 | -61.42054 |  |  |  |  |
|  | LA00788014 | X |  |  | Antigua | 18.11638 | -62.98814 |  |  |  |  |
|  | LA00788023 | X |  |  | Guadeloupe | 16.37752 | -61.47869 |  |  |  |  |
|  | LA00788070 | X |  |  | Saint-Martine (Anguilla) | 18.03007 | -63.09035 |  |  |  |  |
|  | LA00788074 | X | X | X | Gustavia (Anguilla) | 17.91924 | -62.86366 |  |  |  |  |
|  | LA00788085 | X | X | X | Antigua | 18.11638 | -62.98814 |  |  |  |  |
| Mona | MO00782316 | X |  |  | Mona | 18.108216 | -67.935522 |  |  |  |  |
|  | MO00782378 | X |  |  | Mona | 18.08917 | -67.90091 |  |  |  |  |
|  | MO00782668 | X |  |  | Mona | 18.08917 | -67.90091 | Black/wh | 6 | 2 | hap14 |
|  | MO00782688 | X |  |  | Mona | 18.087 | -67.900911 |  |  |  |  |
|  | MO00782695 | X | X | X | Mona | 18.08917 | -67.90091 |  |  |  |  |
|  | MO00784453 | X |  |  | Mona | 18.08917 | -67.90091 |  |  |  |  |
|  | MO00784629 | X |  |  | Mona | 18.08917 | -67.90091 | Black/wh | 6 | 2 | hap14 |
|  | MO00784807 | X | X |  | Mona | 18.08917 | -67.90091 | Black/wh | 6 | 2 | hap36 |
|  | MO00785036 | X |  |  | Mona | 18.08917 | -67.90091 |  |  |  |  |
|  | MO00785469 | X |  | X | Mona | 18.08917 | -67.90091 | Black/wh | 6 | 2 | hap14 |
|  | MO00786845 | X | X | X | Mona | 18.08917 | -67.90091 |  |  |  |  |
|  | MO00786848 | X |  |  | Mona | 18.08917 | -67.90091 |  |  |  |  |
|  | MO00786857 | X |  |  | Mona | 18.08917 | -67.90091 |  |  |  |  |
|  | MO00786886 | X |  |  | Mona | 18.08917 | -67.90091 | Black/wh | 6 | 2 | hap14 |
|  | MO00786909 | X |  |  | Mona | 18.08917 | -67.90091 | Black/wh | 6 | 2 | hap14 |
|  | MO00787057 | X |  |  | Mona | 18.08917 | -67.90091 |  |  |  |  |
| Puerto Rico | PR00392720 | X |  |  | Guanica | 17.97147 | -66.86796 |  |  |  |  |
|  | PR00392750 | X | X | X | El Yunque | 18.32169 | -65.81991 | yellow | 4 | 3 | hap38 |
|  | PR00392800 | X |  | X | Guanica | 17.95387 | -66.84918 |  |  |  |  |
|  | PR00392815 | X | X |  | Guanica | 17.95387 | -66.84918 | yellow | 4 | 2 | hap5 |
|  | PR00392827 | X |  |  | Guanica | 17.95387 | -66.84918 | white | 4 | 3 | hap13 |
|  | PR00392839 | X |  |  | Guanica | 17.95387 | -66.84918 |  |  |  |  |
|  | PR00392841 | X |  |  | Guanica | 17.95387 | -66.84918 |  |  |  |  |
|  | PR00392890 | X |  |  | Guanica | 17.95387 | -66.84918 | Black/wh | 4 | 3 | hap13 |
|  | PR00392898 | X | X | X | Guanica | 17.95387 | -66.84918 | Black/wh | 4 | 3 | hap13 |
|  | PR00392969 | X | X | X | El Yunque | 18.32169 | -65.81991 | yellow | 4 | 3 | hap13 |
|  | PR00782149 | X |  |  | Toro Negro | 18.17298 | -66.4918 | black | 4 | 3 | hap40 |
|  | PR00782234 | X |  |  | Guanica | 17.97147 | -66.86796 |  |  |  |  |
|  | PR00782243 | X |  |  | Guanica | 17.97147 | -66.86796 | white | 4 |  |  |
|  | PR00783338 | X |  |  | Guanica | 18.41437 | -66.72872 | white | 4 | 3 | hap13 |
| TCI | TCI00003904A | X |  |  | Provo | 21.7981 | -72.1707833 | white | 4 | 1 | hap41 |
|  | TCI00004611A | X |  |  | East | 21.7517667 | -71.59665 | black | 4 | 1 | hap41 |
|  | TCI00004648A | X |  |  | Middle | 21.8076 | -71.7010333 | yellow | 4 | 1 | hap41 |
|  | TCI00006129A | X |  |  | Provo | 21.7981 | -72.1707833 | yellow | 4 | 1 | hap42 |
| SEUS | EV00003757 | X |  |  |  | 25.45349 | -80.45919 | white | 6 |  |  |
|  | SU00000263 | X |  |  |  | 33.03907 | -79.56458 | white | 6 |  |  |
|  | SU00000357 | X |  | X |  | 33.03913 | -79.56459 | white | 6 |  |  |
|  | SU00000687 | X |  |  |  | 33.03913 | -79.56459 | white | 6 |  |  |
|  | SU00000715 | X | X | X |  | 33.03907 | -79.56458 | white | 6 |  |  |
|  | SU00000919 | X |  |  |  | 33.03913 | -79.56459 | white | 6 |  |  |
|  | SU00000963 | X |  |  |  | 33.03913 | -79.56459 | white | 6 |  |  |
|  | SU00001014 | X |  |  |  | 33.03907 | -79.56458 | white | 6 |  |  |
|  | SU00001015 | X |  |  |  | 29.62986 | -82.2988 | white | 6 |  |  |
|  | SU00001025 | X |  | X |  | 29.62688 | -82.29878 | white | 6 |  |  |
|  | SU00001045 | X | X | X |  | 33.03907 | -79.56458 | white | 6 |  |  |
|  | SU00001062 | X |  |  |  | 29.62688 | -82.29878 | white | 6 |  |  |
|  | SU00001099 | X | X | X |  | 29.62986 | -82.2988 | white | 6 |  |  |
|  | SU00001123 | X |  |  |  | 29.4776 | -82.55272 | white | 6 |  |  |
|  | SU00001138 | X |  |  |  | 33.03907 | -79.56458 | white | 6 |  |  |
|  | SU00001144 | X | X | X |  | 33.03907 | -79.56458 | white | 6 |  |  |
|  | SU00001234 | X | X | X |  | 33.03907 | -79.56458 | white | 6 |  |  |
|  | SU00001248 | X |  |  |  | 29.6368 | -82.23961 | white | 6 |  |  |
|  | SU00001258 | X | X | X |  | 29.6368 | -82.23961 | white | 6 |  |  |
|  | SU00001339 | X | X |  |  | 29.6368 | -82.23961 | white | 6 |  |  |
| Brazil | SEQ01 | X | X | X | Acre | -9.9817 | -67.811 |  |  |  |  |
|  | SEQ02 | X | X | X | Acre | -9.9817 | -67.811 |  |  |  |  |
|  | SEQ03 | X | X | X | Acre | -9.9817 | -67.811 |  |  |  |  |
|  | SEQ04 | X | X | X | Acre | -9.9817 | -67.811 |  |  |  |  |
|  | SEQ05 | X | X | X | Acre | -9.9817 | -67.811 |  |  |  |  |
|  | SEQ06 | X | X | X | Acre | -9.9817 | -67.811 |  |  |  |  |
|  | SEQ07 | X | X | X | Acre | -9.9817 | -67.811 |  |  |  |  |
|  | SEQ08 | X | X | X | Bahia | -12.5254 | -38.0148 |  |  |  |  |
|  | SEQ09 | X | X | X | Bahia | -12.5254 | -38.0148 |  |  |  |  |
|  | SEQ10 | X | X | X | Bahia | -12.5254 | -38.0148 |  |  |  |  |
|  | SEQ11 | X | X | X | Bahia | -12.5254 | -38.0148 |  |  |  |  |
|  | SEQ12 | X | X | X | Bahia | -12.5254 | -38.0148 |  |  |  |  |
|  | SEQ13 | X | X | X | Bahia | -12.5254 | -38.0148 |  |  |  |  |
|  | SEQ14 | X | X | X | Bahia | -12.5254 | -38.0148 |  |  |  |  |
|  | SEQ15 | X | X | X | Bahia | -12.5254 | -38.0148 |  |  |  |  |
|  | SEQ16 | X | X | X | Bahia | -12.5605 | -41.3859 |  |  |  |  |
|  | SEQ17 | X | X | X | Bahia | -12.5605 | -41.3859 |  |  |  |  |
|  | SEQ18 | X | X | X | Bahia | -12.5605 | -41.3859 |  |  |  |  |
|  | SEQ19 | X | X | X | Bahia | -12.5605 | -41.3859 |  |  |  |  |
|  | SEQ20 | X | X | X | Bahia | -12.5605 | -41.3859 |  |  |  |  |
|  | SEQ21 | X | X | X | Bahia | -12.5605 | -41.3859 |  |  |  |  |
|  | SEQ22 | X | X | X | Bahia | -12.5605 | -41.3859 |  |  |  |  |
|  | SEQ23 | X | X | X | Bahia | -12.5605 | -41.3859 |  |  |  |  |
|  | SEQ24 | X | X | X | Sao Paulo | -22.8189 | -47.0698 |  |  |  |  |
| Colombia | SEQ100 | X | X | X | Antioquia | 6.2079 | -75.5699 |  |  |  |  |
|  | SEQ101 | X | X | X | Choco | 6.3853 | -77.3999 |  |  |  |  |
|  | SEQ102 | X | X | X | Sucre | 9.5932 | -75.571 |  |  |  |  |
|  | SEQ103 | X | X | X | Sucre | 9.5932 | -75.571 |  |  |  |  |
|  | SEQ104 | X | X | X | Sucre | 9.5932 | -75.571 |  |  |  |  |
|  | SEQ105 | X | X | X | Sucre | 9.5932 | -75.571 |  |  |  |  |
|  | SEQ25 | X | X | X | Tolima | 4.4281 | -75.2129 |  |  |  |  |
|  | SEQ26 | X | X | X | Tolima | 4.4281 | -75.2129 |  |  |  |  |
|  | SEQ27 | X | X | X | Tolima | 4.4281 | -75.2129 |  |  |  |  |
|  | SEQ28 | X | X | X | Tolima | 4.4281 | -75.2129 |  |  |  |  |
|  | SEQ29 | X | X | X | Tolima | 4.4281 | -75.2129 |  |  |  |  |
|  | SEQ30 | X | X | X | Tolima | 4.4281 | -75.2129 |  |  |  |  |
|  | SEQ31 | X | X | X | Tolima | 4.4444 | -75.1916 |  |  |  |  |
|  | SEQ32 | X | X | X | Tolima | 4.4444 | -75.1916 |  |  |  |  |
|  | SEQ33 | X | X | X | Tolima | 4.4444 | -75.1916 |  |  |  |  |
|  | SEQ34 | X | X | X | Tolima | 4.4444 | -75.1916 |  |  |  |  |
|  | SEQ35 | X | X | X | Tolima | 4.4444 | -75.1916 |  |  |  |  |
|  | SEQ36 | X | X | X | Tolima | 5.0023 | -74.9077 |  |  |  |  |
|  | SEQ37 | X | X | X | Tolima | 5.0023 | -74.9077 |  |  |  |  |
|  | SEQ38 | X | X | X | Tolima | 5.0023 | -74.9077 |  |  |  |  |
|  | SEQ39 | X | X | X | Tolima | 5.0023 | -74.9077 |  |  |  |  |
|  | SEQ40 | X | X | X | Guajira | 11.2516 | -73.5584 |  |  |  |  |
|  | SEQ41 | X | X | X | Guajira | 11.2516 | -73.5584 |  |  |  |  |
|  | SEQ42 | X | X | X | Guajira | 11.2516 | -73.5584 |  |  |  |  |
|  | SEQ43 | X | X | X | Guajira | 11.2516 | -73.5584 |  |  |  |  |
|  | SEQ44 | X | X | X | Valle del Cauca | 3.5678 | -76.5739 |  |  |  |  |
|  | SEQ45 | X | X | X | Valle del Cauca | 3.5678 | -76.5739 |  |  |  |  |
|  | SEQ46 | X | X | X | Valle del Cauca | 3.5678 | -76.5739 |  |  |  |  |
|  | SEQ47 | X | X | X | Valle del Cauca | 3.5678 | -76.5739 |  |  |  |  |
|  | SEQ48 | X | X | X | Valle del Cauca | 3.5678 | -76.5739 |  |  |  |  |
|  | SEQ49 | X | X | X | Valle del Cauca | 3.5678 | -76.5739 |  |  |  |  |
|  | SEQ50 | X | X | X | Valle del Cauca | 3.5678 | -76.5739 |  |  |  |  |
|  | SEQ51 | X | X | X | Valle del Cauca | 3.5678 | -76.5739 |  |  |  |  |
|  | SEQ52 | X | X | X | Meta | 4.0727 | -73.5869 |  |  |  |  |
|  | SEQ53 | X | X | X | Meta | 4.0727 | -73.5869 |  |  |  |  |
|  | SEQ54 | X | X | X | Meta | 4.0727 | -73.5869 |  |  |  |  |
|  | SEQ55 | X | X | X | Meta | 4.0727 | -73.5845 |  |  |  |  |
|  | SEQ56 | X | X | X | Meta | 4.0727 | -73.5845 |  |  |  |  |
|  | SEQ57 | X | X | X | Meta | 4.1644 | -73.5813 |  |  |  |  |
|  | SEQ58 | X | X | X | Meta | 4.1644 | -73.5813 |  |  |  |  |
|  | SEQ59 | X | X | X | Meta | 4.1644 | -73.5813 |  |  |  |  |
|  | SEQ60 | X | X | X | Meta | 4.1644 | -73.5821 |  |  |  |  |
|  | SEQ61 | X | X | X | Meta | 4.1644 | -73.6416 |  |  |  |  |
|  | SEQ62 | X | X | X | Meta | 4.0671 | -73.7114 |  |  |  |  |
|  | SEQ63 | X | X | X | Meta | 4.0671 | -73.7114 |  |  |  |  |
|  | SEQ64 | X | X | X | Meta | 4.0671 | -73.7114 |  |  |  |  |
|  | SEQ65 | X | X | X | Meta | 4.0671 | -73.7114 |  |  |  |  |
|  | SEQ66 | X | X | X | Meta | 4.0671 | -73.7114 |  |  |  |  |
|  | SEQ67 | X | X | X | Meta | 3.6128 | -73.7913 |  |  |  |  |
|  | SEQ68 | X | X | X | Meta | 3.6128 | -73.7913 |  |  |  |  |
|  | SEQ69 | X | X | X | Meta | 3.6166 | -73.799 |  |  |  |  |
|  | SEQ70 | X | X | X | Meta | 3.6128 | -73.7913 |  |  |  |  |
|  | SEQ71 | X | X | X | Meta | 4.1758 | -73.6813 |  |  |  |  |
|  | SEQ72 | X | X | X | Meta | 4.1758 | -73.6813 |  |  |  |  |
|  | SEQ73 | X | X | X | Meta | 4.1758 | -73.6813 |  |  |  |  |
|  | SEQ74 | X | X | X | Meta | 4.1758 | -73.6813 |  |  |  |  |
|  | SEQ75 | X | X | X | Meta | 4.1758 | -73.6813 |  |  |  |  |
|  | SEQ76 | X | X | X | Quindio | 4.6375 | -75.5872 |  |  |  |  |
|  | SEQ77 | X | X | X | Quindio | 4.6375 | -75.5872 |  |  |  |  |
|  | SEQ78 | X | X | X | Quindio | 4.6375 | -75.5872 |  |  |  |  |
|  | SEQ79 | X | X | X | Quindio | 4.6375 | -75.5872 |  |  |  |  |
|  | SEQ80 | X | X | X | Quindio | 4.6375 | -75.5872 |  |  |  |  |
|  | SEQ81 | X | X | X | Quindio | 4.6375 | -75.5872 |  |  |  |  |
|  | SEQ82 | X | X | X | Quindio | 4.6375 | -75.5872 |  |  |  |  |
|  | SEQ83 | X | X | X | Quindio | 4.6375 | -75.5872 |  |  |  |  |
|  | SEQ84 | X | X | X | Valle del Cauca | 3.9833 | -77.35 |  |  |  |  |
|  | SEQ85 | X | X | X | Valle del Cauca | 3.9833 | -77.35 |  |  |  |  |
|  | SEQ86 | X | X | X | Valle del Cauca | 4.1023 | -77.4912 |  |  |  |  |
|  | SEQ87 | X | X | X | Valle del Cauca | 4.1023 | -77.4912 |  |  |  |  |
|  | SEQ88 | X | X | X | Valle del Cauca | 4.1023 | -77.4912 |  |  |  |  |
|  | SEQ89 | X | X | X | Valle del Cauca | 4.1023 | -77.4912 |  |  |  |  |
|  | SEQ90 | X | X | X | Valle del Cauca | 4.1023 | -77.4912 |  |  |  |  |
|  | SEQ91 | X | X | X | Valle del Cauca | 4.1023 | -77.4912 |  |  |  |  |
|  | SEQ92 | X | X | X | Valle del Cauca | 4.1023 | -77.4912 |  |  |  |  |
|  | SEQ93 | X | X | X | Amazonas | -4.1813 | -69.952 |  |  |  |  |
|  | SEQ94 | X | X | X | Santander | 7.1426 | -73.1196 |  |  |  |  |
|  | SEQ95 | X | X | X | Santander | 7.1426 | -73.1196 |  |  |  |  |
|  | SEQ96 | X | X | X | Santander | 7.1426 | -73.1196 |  |  |  |  |
|  | SEQ97 | X | X | X | Valle del Cauca | 4.1786 | -76.2058 |  |  |  |  |
|  | SEQ98 | X | X | X | Valle del Cauca | 4.1786 | -76.2058 |  |  |  |  |
|  | SEQ99 | X | X | X | Antioquia | 6.2079 | -75.5699 |  |  |  |  |
| Outgroups | Genbank accession number | *COI* | *16S* | *ITS2* |  |  |  |  |  |  |  |
| G. kuhlii | KU055747.1 |  | X |  |  |  |  |  |  |  |  |
| G. kuhlii | AB910447.1 | X |  |  |  |  |  |  |  |  |  |
| G. sp | DQ127354.1 |  |  |  |  |  |  |  |  |  |  |
| M.millitaris | KJ157294.1 | X |  |  |  |  |  |  |  |  |  |
| M.millitaris | KJ157025.1 |  | X |  |  |  |  |  |  |  |  |
| M.millitaris | KJ157133.1 |  |  | X |  |  |  |  |  |  |  |
| M. gracilis | KJ157195.1 |  |  | X |  |  |  |  |  |  |  |
| Argiope butchko | KX8170771 | X |  |  |  |  |  |  |  |  |  |
| T. grallator | JN863349.1 | X |  |  |  |  |  |  |  |  |  |
| Z.nearctica | KU876530.1 | X |  |  |  |  |  |  |  |  |  |
