# Supplementary material for: Phylogeography of the widespread Caribbean spiny orb weaver Gasteracantha cancriformis
Source: PeerJ. 2020 Apr 30;8:e8976. doi: 10.7717/peerj.8976 (PMC7196328; doi:10.7717/peerj.8976)
Supplement: Supplemental Information 6 [file peerj-08-8976-s006.docx]

| Gene | Primer 1 | Primer 2 | Annealing Temp. (°C) | Fragment Length (bp) | Extension time (s) |
| --- | --- | --- | --- | --- | --- |
| Cytochrome Oxidase 1 – CO1 | LCO11490 | C1-N-2776 | 48-50 | 1250 | 90 |
| 16S | 16SA/12261 | 16SB | 48 | 400-500 | 65 |
| ITS2 | ITS4 | ITS5.8 | 47 | 350-500 | 72 |
