# Supplementary material for: Phylogeography of the widespread Caribbean spiny orb weaver Gasteracantha cancriformis
Source: PeerJ. 2020 Apr 30;8:e8976. doi: 10.7717/peerj.8976 (PMC7196328; doi:10.7717/peerj.8976)
Supplement: Supplemental Information 7 [file peerj-08-8976-s007.docx]

|  | Cuba | Hispaniola | Jamaica | Lesser  Antilles | Mona | Puerto  Rico | Turks and  Caicos |
| --- | --- | --- | --- | --- | --- | --- | --- |
| Cuba |  | 0.74771 | 4.30177 | 0.39041 | 0.13475 | 0.71967 | 5.92708 |
| Hispaniola | 0.40073 |  | 0.4078 | 0.36778 | 0.89961 | 0.69584 | 0.40218 |
| Jamaica | 0.10413* | 0.55078* |  | 0.2843 | 0.18254 | 0.39542 | ∞ |
| Lesser  Antilles | 0.56154* | 0.57619* | 0.63751* |  | 0.14848 | 1.73414 | 0.20738 |
| Mona | 0.78772* | 0.35724* | 0.73256* | 0.77103* |  | 0.22821 | 0.03456 |
| Puerto  Rico | 0.40995* | 0.41812* | 0.5584* | 0.2238* | 0.68661* |  | 0.34219 |
| Turks and Caicos | 0.0778 | 0.55421* | -0.00279 | 0.70683* | 0.93536* | 0.59369* |  |
