# Supplementary material for: Phylogeography of the widespread Caribbean spiny orb weaver Gasteracantha cancriformis
Source: PeerJ. 2020 Apr 30;8:e8976. doi: 10.7717/peerj.8976 (PMC7196328; doi:10.7717/peerj.8976)
Supplement: Supplemental Information 8 [file peerj-08-8976-s008.docx]

| Pop 1 | Pop 2 | Hs | Ks | Kxy | G_ST_ | DeltaSt | GammaSt | N_ST_ | *F*ST | *d_XY_* | Da |
| --- | --- | --- | --- | --- | --- | --- | --- | --- | --- | --- | --- |
| Cuba | SEUS | 0.86667 | 3.17333 | 3.41667 | 0.03473 | 0.00072 | 0.13194 | 0.01452 | 0.01463 | 0.00646 | 0.00009 |
| Cuba | His | 0.8253 | 2.86992 | 5.22973 | 0.06048 | 0.00076 | 0.1298 | 0.32728 | 0.32644 | 0.00989 | 0.00323 |
| Cuba | Jamaica | 0.89105 | 3.34942 | 4 | 0.04946 | 0.00046 | 0.07429 | 0.0646 | 0.06425 | 0.00756 | 0.00049 |
| Cuba | LA | 0.88043 | 2.63147 | 6.22917 | 0.0523 | 0.00161 | 0.26258 | 0.46544 | 0.46372 | 0.01178 | 0.00546 |
| Cuba | Mona | 0.23438 | 0.96667 | 6.4375 | 0.33101 | 0.00288 | 0.67163 | 0.65473 | 0.65372 | 0.01217 | 0.00796 |
| Cuba | PR | 0.83987 | 3.65527 | 6.32143 | 0.05889 | 0.00202 | 0.24892 | 0.38461 | 0.38346 | 0.01195 | 0.00458 |
| Cuba | TCI | 0.75 | 2.41667 | 2.625 | 0.14286 | 0.00077 | 0.1831 | 0.0783 | 0.07937 | 0.00496 | 0.00039 |
| SEUS | His | 0.81374 | 2.66822 | 4.82883 | 0.07576 | 0.00114 | 0.19119 | 0.47142 | 0.47071 | 0.00913 | 0.0043 |
| SEUS | Jamaica | 0.86459 | 2.97895 | 4.48333 | 0.07572 | 0.00133 | 0.20315 | 0.38183 | 0.38075 | 0.00848 | 0.00323 |
| SEUS | LA | 0.85886 | 2.35826 | 5.22222 | 0.07454 | 0.00187 | 0.31059 | 0.54683 | 0.54542 | 0.00987 | 0.00538 |
| SEUS | Mona | 0.275 | 0.74545 | 5.60417 | 0.41234 | 0.00341 | 0.74087 | 0.77544 | 0.77472 | 0.01059 | 0.00821 |
| SEUS | PR | 0.80989 | 3.14308 | 5.42857 | 0.09902 | 0.00224 | 0.29368 | 0.46078 | 0.46012 | 0.01026 | 0.00472 |
| SEUS | TCI | 0.7 | 1.64 | 3.5 | 0.20078 | 0.0021 | 0.45122 | 0.58619 | 0.58571 | 0.00662 | 0.00388 |
| His | Jamaica | 0.83693 | 2.86642 | 6.40405 | 0.07821 | 0.00309 | 0.37159 | 0.54352 | 0.54214 | 0.01211 | 0.00656 |
| His | LA | 0.83625 | 2.56854 | 5.92568 | 0.08313 | 0.00314 | 0.40067 | 0.57435 | 0.57308 | 0.0112 | 0.00642 |
| His | Mona | 0.61808 | 1.93082 | 3.07939 | 0.26139 | 0.00136 | 0.27644 | 0.53912 | 0.5394 | 0.00582 | 0.00314 |
| His | PR | 0.81477 | 2.91755 | 5.14093 | 0.05991 | 0.00167 | 0.23979 | 0.40034 | 0.3996 | 0.00972 | 0.00388 |
| His | TCI | 0.79827 | 2.49594 | 5.02027 | 0.1085 | 0.00117 | 0.20376 | 0.68055 | 0.68013 | 0.00949 | 0.00645 |
| Jamaica | LA | 0.87379 | 2.71365 | 7.3125 | 0.06677 | 0.0044 | 0.47324 | 0.62588 | 0.6239 | 0.01382 | 0.00862 |
| Jamaica | Mona | 0.5491 | 1.80702 | 7.6875 | 0.31156 | 0.00572 | 0.63827 | 0.7879 | 0.78682 | 0.01453 | 0.01143 |
| Jamaica | PR | 0.85264 | 3.27983 | 7.4 | 0.08129 | 0.00393 | 0.40278 | 0.55471 | 0.5531 | 0.01399 | 0.00774 |
| Jamaica | TCI | 0.84105 | 2.71053 | 2.1 | 0.12209 | 0.00022 | 0.04312 | 0.12928 | 0.13033 | 0.00397 | 0.00052 |
| LA | Mona | 0.58001 | 1.4587 | 7.1875 | 0.29731 | 0.00545 | 0.67353 | 0.82889 | 0.82798 | 0.01359 | 0.01125 |
| LA | PR | 0.84967 | 2.75814 | 3.67857 | 0.06199 | 0.00083 | 0.14461 | 0.21064 | 0.21038 | 0.00695 | 0.00146 |
| LA | TCI | 0.83877 | 2.08385 | 6.5 | 0.11639 | 0.0024 | 0.39059 | 0.78201 | 0.78094 | 0.01229 | 0.0096 |
| Mona | PR | 0.44262 | 1.68205 | 6.17857 | 0.37205 | 0.00425 | 0.58978 | 0.71039 | 0.70976 | 0.01168 | 0.00829 |
| Mona | TCI | 0.17188 | 0.2 | 6.6875 | 0.5301 | 0.0039 | 0.92433 | 0.95361 | 0.95327 | 0.01264 | 0.01205 |
| PR | TCI | 0.76845 | 2.80342 | 6.5 | 0.15572 | 0.00307 | 0.38636 | 0.69586 | 0.69527 | 0.01229 | 0.00854 |
